# Reversible Bilateral Vision Loss: An Unusual Presentation of Wernicke-Korsakoff Syndrome

**DOI:** 10.7759/cureus.64613

**Published:** 2024-07-15

**Authors:** Emily Guagliardo, Divya Singh, Jasmine Thakkar, Wilson Rodriguez, Francesca Pastrana, Hanan Qaqish, Pratap Chand

**Affiliations:** 1 Neurology, Saint Louis University School of Medicine, St. Louis, USA

**Keywords:** wernicke-korsakoff syndrome, optic perineuritis, anti-mog antibody, retinal ganglion cell neuronopathy, optic neuropathy, nutritional deficiency, neuropathy, thiamine, vision loss

## Abstract

Neuro-ophthalmic manifestations of Wernicke encephalopathy (WE) are uncommon and vary from nystagmus, oculomotor palsies, anisocoria, and optic disc edema to vision loss. We describe a case of a 53-year-old woman presenting with subacute bilateral painless vision decline, lower-extremities weakness with impaired ambulation, headache, and abdominal pain. Neurological examination was pertinent for confabulation, bilateral decreased visual acuity with an absent blink to threat, absent afferent pupillary defect and fundus abnormalities, and significant allodynia in bilateral lower extremities. Besides elevated inflammatory marker with an erythrocyte sedimentation rate (ESR) of 130 mm/hr, her infectious, autoimmune, paraneoplastic, and neuromyelitis optica work-up was overall unremarkable. Brain MRI showed abnormal fluid-attenuated inversion recovery (FLAIR) signaling in bilateral mammillary bodies and around periaqueductal gray matter concerning WE. Due to concerns of Wernicke-Korsakoff syndrome (WKS), parenteral high-dose thiamine was initiated with significant clinical improvement. The patient was also later found to have a positive anti-myelin oligodendrocyte glycoprotein (MOG) antibody, which was deemed false positive given the atypical phenotype and symptomatic improvement with thiamine supplementation. This case encourages the consideration of vision loss as a manifestation of WKS, especially in patients who have risk factors. Testing serum levels of thiamine is strongly encouraged; however, initiating empiric treatment is advocated for high clinical suspicion due to its reversible nature and minimal risk for side effects.

## Introduction

Wernicke encephalopathy (WE), first described in 1881 by Carl Wernicke as a triad of altered mental status, ataxia, and oculomotor dysfunction [1,2], is a well-known neuropsychiatric disorder associated with thiamine deficiency. If left untreated, it can result in amnesia and confabulation in its chronic phase collectively constituting Wernicke-Korsakoff syndrome (WKS) [1,2]. Chronic alcohol use is often associated with this nutritional deficiency either from reduced intake or impaired utilization resulting in ultrastructural changes in myelin sheath and, thus possibly reversible, white matter degeneration [3]. Clinically, encephalopathy remains its most common manifestation. Ocular findings when reported include nystagmus, gaze palsy, internuclear ophthalmoplegia, anisocoria, miosis, optic disc edema, and retinal hemorrhages [1]. Vision loss is one of its uncommon neuro-ophthalmologic manifestations, which we describe here in a 53-year-old female who underwent a myriad of investigations before the final diagnosis of WKS and its treatment.

This article was previously presented as a meeting abstract at the American Academy of Neurology 2024 Annual Meeting on April 14, 2024.

## Case presentation

A 53-year-old woman with a history of psoriatic arthritis, latent syphilis treated with penicillin, polysubstance use inclusive of cocaine and marijuana, and chronic alcoholism (one pint of vodka daily for several years) presented with subacute progressively worsening bilateral painless vision loss over three weeks, bilateral lower-extremity weakness, and paresthesia. She also endorsed auditory hallucinations, abdominal pain, headaches, and difficulty walking. Memory decline over the last few months was noted by the family impacting her compliance with medications.

**Figure 1:**
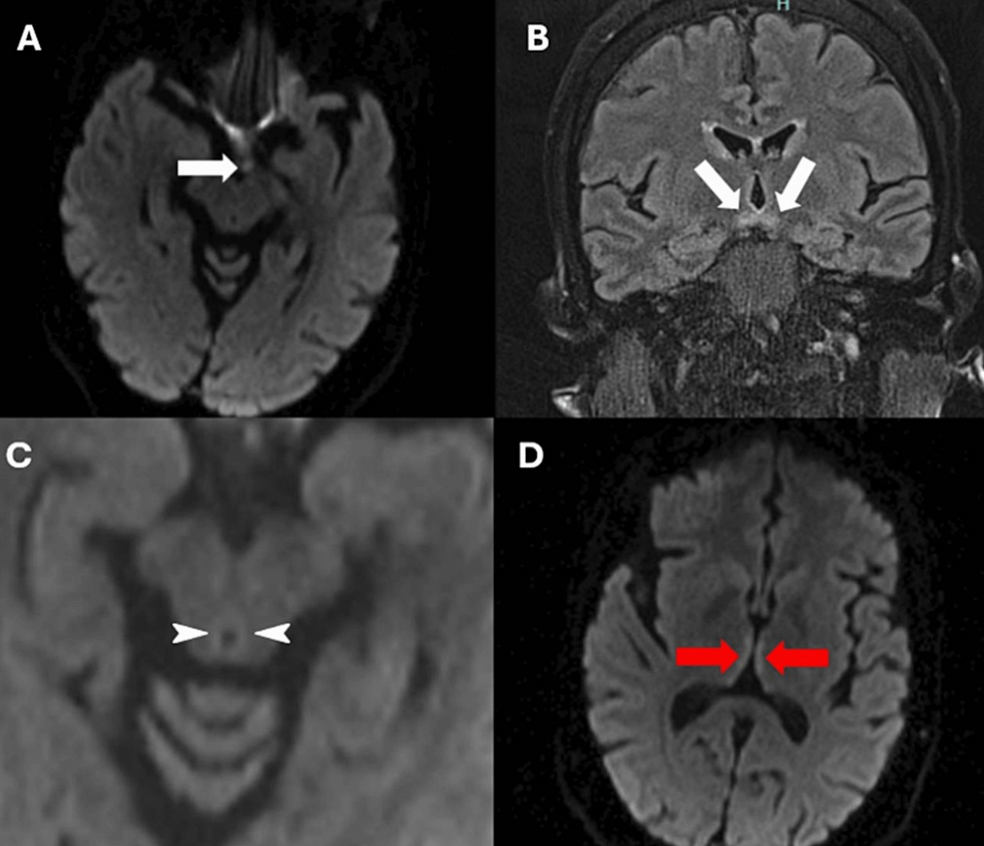
Brain MRI with and without contrast. A: Axial diffusion-weighted imaging (DWI) sequence showing diffusion restriction in mamillary bodies (white arrows). B: Coronal flair sequence showing hyperintensity in mamillary bodies (white arrows). C: Axial DWI sequence showing diffusion restriction in the periaqueductal gray matter (white arrows head). D: DWI showing diffusion restriction in the medial thalamic nuclei (red arrows).

Neurological examination was pertinent for confabulation, restricted horizontal gaze bilaterally, significantly reduced bilateral visual acuity with an absent blink to threat and loss of color perception, decreased vibration, allodynia, and hyporeflexia in the lower extremities. The examination was negative for afferent pupillary defect (APD) and fundus abnormalities. Initial work-up demonstrated elevated inflammatory markers with an erythrocyte sedimentation rate (ESR) of 130 mm/hr, and positive treponema pallidum antibody but negative rapid plasma reagin (RPR). Due to concerns of serodiscordant neurosyphilis, a lumbar puncture was done, which only showed a borderline high protein of 45 mg/dl (normal 15-45 mg/dL) and later a non-reactive venereal disease research laboratory (VDRL) test. Infectious, nutritional/metabolic, autoimmune, paraneoplastic, and demyelinating illness work-up was done, which returned positive for folate deficiency. Oral daily folate supplements of 1 mg were initiated, but no immediate improvement was endorsed. Brain magnetic resonance imaging (MRI), limited from motion artifacts, was unremarkable. Electromyography with nerve conduction was pursued due to allodynia, which showed diffuse sensorimotor axonal polyneuropathy. MR angiography of the head and neck performed later did not have signs of vasculopathy. Repeat brain MRI with and without contrast demonstrated diffusion restriction in bilateral mammillary bodies, around the periaqueductal gray matter, and hyperintensity in medial thalami concerning for WKS (Figure 1). MRI of the orbits showed minimal enhancement surrounding optic nerves suggesting retrobulbar optic perineuritis.

High-dose parenteral thiamine 500 mg daily was, therefore, initiated for three days with significant improvement in both vision and allodynia. The diagnosis of WKS was then established, and maintenance oral thiamine supplementation of 100 mg daily continued afterward. Serum thiamine level, obtained after starting therapy, was 166 nmol/L (70-180 nmol/L normal range). The patient was eventually discharged to acute rehabilitation due to persistent therapy needs.

A week after discharge, her serum myelin oligodendrocyte glycoprotein (MOG) antibody returned positive. She was followed in a neuro-ophthalmology clinic, where no disc edema was elicited; rather, they noticed in optical coherence tomography diffuse ganglion cell complex thinning bilaterally with preserved retinal nerve fiber layer thickness suggesting optic atrophy secondary to nutritional deficiency.

## Discussion

WE, as aforementioned, encompasses the triad of altered mentation, ataxia, and oculomotor dysfunction [1,2]. However, this classic triad is not always present. It is estimated to be seen in only 16.0-23.5% of patients [4,5]. Risk factors include chronic alcohol use, malnutrition, malabsorption, systemic disease, certain medications, and dietary loss through severe vomiting or diarrhea [6]. In our case, the patient with a risk factor of chronic alcoholism presented with classic symptoms of ophthalmoplegia, ataxia, and confusion accompanied by confabulation, along with several atypical findings. Some underrecognized features of WE/WKS inclusive of vision loss, peripheral neuropathy, hallucinations, autonomic symptoms of headache, and abdominal pain were also present [2]. The vision loss to the extent of near blindness evident in our patient could be attributed to optic neuropathy from thiamine deficiency.

The optic perineuritis seen in our patient could be hypothesized to be secondary to necrosis of nerve cells and myelinated structures, possibly leading to inflammation within the optic nerve itself [7]. While it can have several causes including autoimmune, demyelinating, or infectious, the clinical improvement in our case immediately after administering high-dose parenteral thiamine supports the primary etiology of thiamine deficiency. In addition, the clinical presentation with subacute onset, absent optic disc edema and APD, and minimal contrast enhancement on imaging was incongruent with MOG-associated disease. It is important to note that anti-MOG antibodies can have high false positivity, which is probably the case with our patient particularly given her atypical phenotype [8].

Similar presentations of vision loss in WE have been reported, where if it is not presenting, it could be a primary symptom of thiamine deficiency [9-11]. Interestingly, the differences in these cases are in the pathophysiology of vision loss, such as optic neuropathy with disc edema [9], peripapillary retinal nerve fiber layer infarcts [10], and thickened and telangiectatic peripapillary nerve fiber layer with retinal hemorrhages [11]. The retinal ganglion cell neuronopathy found in the outpatient examination a few months later in our patient was consistent with optic atrophy due to nutritional deficiencies like folate and thiamine.

Furthermore, the concurrent picture of dry beriberi is also possible given the sensorimotor axonal polyneuropathy and autonomic symptoms of abdominal pain and headache [1,12]. However, this further highlights the vast variety of non-specific symptoms that can coexist in WE.

While WE and WKS are clinical diagnoses, workup includes serum and urine thiamine assays, which are not very reliable, and measurement of thiamine pyrophosphate or erythrocyte transketolase whose precise sensitivity and specificity are not established. The absence of these measurements could be a limitation to our case. However, repeat MRI findings and clinical improvement with supplementation supported our diagnosis. MRI findings in WE/WKS include T2 hyperintensities in the thalamus, hypothalamus, mammillary bodies, periaqueductal region, the floor of the fourth ventricle, and midline cerebellum [6], but they have a sensitivity of 53% and specificity of 93% [6,13]. The low sensitivity could explain why the initial MRI of our patient did not reveal any abnormalities, thus reiterating the necessity to have high suspicion for WE despite normal imaging.

## Conclusions

Prompt diagnosis of WKS is imperative due to the irreversible cognitive impairment and possible fatal nature of the disease. Atypical features like vision loss should suggest WKS as differential, particularly in high-risk individuals. Testing serum thiamine levels is recommended in this population. However, initiating empiric treatment is advocated for high clinical suspicion due to its reversible nature, minimal risk for side effects, and potential for permanent neurologic deficits if left untreated.
